# High dose Erythropoietin increases Brain Tissue Oxygen Tension in Severe Vasospasm after Subarachnoid Hemorrhage

**DOI:** 10.1186/1471-2377-12-32

**Published:** 2012-06-06

**Authors:** Raimund Helbok, Ehab Shaker, Ronny Beer, Andreas Chemelli, Martin Sojer, Florian Sohm, Gregor Broessner, Peter Lackner, Monika Beck, Alexandra Zangerle, Bettina Pfausler, Claudius Thome, Erich Schmutzhard

**Affiliations:** 1 Neurological Intensive Care Unit, Department of Neurology, Innsbruck Medical University, Innsbruck, Austria; 2 Department of Neurology, Cairo University, Cairo, Egypt; 3 Department of Radiology, Innsbruck Medical University, Innsbruck, Austria; 4 Department of Neurosurgery, Innsbruck Medical University, Innsbruck, Austria

**Keywords:** Cerebral microdialysis, Erythropoietin, Multimodality monitoring, Subarachnoid hemorrhage

## Abstract

**Background:**

Vasospasm-related delayed cerebral ischemia (DCI) significantly impacts on outcome after aneurysmal subarachnoid hemorrhage (SAH). Erythropoietin (EPO) may reduce the severity of cerebral vasospasm and improve outcome, however, underlying mechanisms are incompletely understood. In this study, the authors aimed to investigate the effect of EPO on cerebral metabolism and brain tissue oxygen tension (P_b_tO_2_).

**Methods:**

Seven consecutive poor grade SAH patients with multimodal neuromonitoring (MM) received systemic EPO therapy (30.000 IU per day for 3 consecutive days) for severe cerebral vasospasm. Cerebral perfusion pressure (CPP), mean arterial blood pressure (MAP), intracranial pressure (ICP), P_b_tO_2_ and brain metabolic changes were analyzed during the next 24 hours after each dose given. Statistical analysis was performed with a mixed effects model.

**Results:**

A total of 22 interventions were analyzed. Median age was 47 years (32–68) and 86 % were female. Three patients (38 %) developed DCI. MAP decreased 2 hours after intervention (P < 0.04) without significantly affecting CPP and ICP. P_b_tO_2_ significantly increased over time (P < 0.05) to a maximum of 7 ± 4 mmHg increase 16 hours after infusion. Brain metabolic parameters did not change over time.

**Conclusions:**

EPO increases P_b_tO_2_ in poor grade SAH patients with severe cerebral vasospasm. The effect on outcome needs further investigation.

## Background

Vasospasm-related delayed cerebral ischemia (DCI) is common and significantly impacts on outcome after aneurysmal subarachnoid hemorrhage (SAH). [1] Underlying mechanisms including inflammation, oxidative distress and apoptosis may lead to secondary brain tissue damage. [2] Strategies to prevent and treat cerebral vasospasm include hyperdynamic therapy and calcium channel blockers, however still remain suboptimal. [1] Other prophylactic agents such as endothelin A receptor antagonists were effective in experimental and animal settings but failed to improve outcome in human trials [3-6]. There is some evidence that acute erythropoietin (EPO) treatment may reduce the severity of cerebral vasospasm and eventually improve outcome in SAH patients [7,8]. Underlying mechanisms extend far beyond erythropoiesis: EPO may enhance neurogenesis, decrease inflammation and inhibit apoptosis especially in the damaged brain where EPO receptors are highly expressed [9,10]. Timing of EPO treatment in the early phase of SAH may be crucial [7,8], however, the acute effect of EPO on brain homeostasis in severe cerebral vasospasm has not been elucidated so far.

In this study we sought to describe the effect of EPO treatment on cerebral perfusion pressure (CPP), brain metabolism and brain tissue oxygen tension (P_b_tO_2_) in SAH patients with severe cerebral vasospasm.

## Methods

### Patient selection and data collection

Between April 2010 and March 2011 seven consecutive poor grade SAH patients with multimodal neuromonitoring (MM) receiving erythropoietin as compassionate treatment for severe cerebral vasospasm were studied. All patients were admitted to the Neurological Intensive Care Unit at Innsbruck Medical University. The clinical care for SAH patients conforms to guidelines set forth by the American Heart Association [1]. All patients received concomitant statin therapy and were on midazolame, sufentanil and/ or ketamine continuous infusions at the days of intervention. Severe vasospasm was defined by mean transcranial doppler (TCD) velocity >180 cm/sec and a Lindegaard ratio >3 when conventional treatment (continuous intravenous nimodipine application and hemodynamic augmentation with CPP target >80 mmHg) failed. DCI was defined as appearance of new infarction on CT that was judged by an independent radiologist to be attributable to cerebral vasospasm.

### Intervention

EPO (Epoetin alfa, Erypo®, Janssen-Cilag Pharma, Vienna, Austria) 30.000 IU diluted to 50 ml of normal saline was administered as infusion over 30 min every day for 3 consecutive days immediately after severe vasospasm was diagnosed. The decision to start the intervention was made by the neurointensivist in charge (ES, BP, RB, RH). In one patient a single dose was given, another patient received two sets of interventions at intervals of 7 days, leading to 22 interventions analyzed.

### Neuromonitoring, data collection and ethical approval

Based on the clinical and imaging criteria, the patient underwent monitoring of cerebral metabolism, brain tissue oxygenation (PbtO2), and ICP according to local institutional protocol which is in compliance with the Helsinki Declaration and has been approved by the local ethics committee (UN3898 285/4.8). Written informed consent was obtained according to federal regulations. Through a right frontal burr hole, a triple-lumen bolt was affixed to insert a Licox Clark-type probe (Integra Licox Brain Oxygen Monitoring, Integra NeuroSciences, Ratingen, Germany) to measure PbtO2 and an ICP parenchymal probe (NEUROVENT_P-TEMP, Raumedic, Münchberg, Germany). In addition, a high cut-off brain microdialysis catheter (CMA 71, Dipylon Medical, Solna, Sweden) was tunneled and inserted into the brain parenchyma for hourly assessment of brain metabolism. Isotonic perfusion fluid (Perfusion Fluid CNS, Dipylon Medical) was pumped through the system at a flow rate of 0.3 μl/min. Hourly samples were analyzed with CMA 600 Microdialysis Analyzer (CMA/Microdialysis, Solna, Sweden*)* for cerebral extracellular glucose, pyruvate, and lactate concentrations. At least 1 h passed after the insertion of the probe and the start of the sampling, to allow for normalization of changes due to probe insertion. The location of the monitoring catheters in the white matter of the right frontal lobe was confirmed by brain CT scan immediately after the procedure. All continuously measured parameters were saved on a 3 min average interval using our patient data management system (Centricity* Critical Care 7.0 SP2, GE Healthcare Information Technologies, Dornstadt, Germany). Transcranial Doppler sonography was performed using the DWL Doppler-Box system *(*Compumedics, Singen Germany). Data on CPP, ICP and MAP were available during all interventions, P_b_tO_2_ and microdialysis in 14 and 18 observations, respectively.

### *Statistic*

Continuous variables were assessed for normality. Normally distributed data were reported as mean and standard error of mean, nonparametric data as median and interquartile range (IQR), unless indicated otherwise. Categorical variables were reported as count and proportions in each group. Hourly recorded brain metabolic parameters were averaged for 6 hours episodes, and continuously recorded parameters (P_b_tO_2_, CPP, MAP, ICP) for 2 hours episodes. Baseline values were calculated accordingly and changes from baseline were analyzed: multiple observations per subject were handled by using generalized estimating equations with an autoregressive working correlation matrix. For all tests, significance was set at P < 0.05. All analyses were performed with SPSS V19.0 (SPSS Inc., Chicago, Illinois).

## Results

### **General characteristics and outcome**

Baseline characteristics are described in the Table 1. Median patient age was 47 years (IQR:32–68) and six of seven were female. None of the patients had a history of malignancy or thrombembolism and no thromboembolic events occurred during hospitalization. All patients developed severe vasospasm in the anterior circulation of the aneurysm site and received Erythropoietin (EPO) at a median of 9 days (IQR:6–12) after ictus. None of the patients died during hospitalization and 3 patients (38 %) developed DCI with evidence of new cerebral infarctions at the site of the ruptured aneurysm distant (>3 cm) to the neuromonitoring probes. In these patients cranial CT scan had been performed after the observation period.

**Table 1:** Characteristics of EPO patient cohort


**Patients**	**Age**	**Sex**	**Admission H&H**	**Modified Fisher**	**SAH sum-score**	**IVH sum-score**	**Infarction on admission CT**	**ICH on admission CT**	
1	53	F	4	3	15	1	no	yes	
2a	40	F	5	1	12	0	no	yes	
2b	40	F	5	1	12	0	no	yes	
3	69	F	4	3	25	8	no	no	
4	32	F	5	4	18	3	no	no	
5	29	M	5	4	14	4	no	no	
6	68	F	5	3	30	0	no	no	
7	47	F	3	4	18	4	no	no	
	**Aneurysm Location**	**Monitoring location**	**1 = coiling 2 = clipping**	**Admission Glucose (mg/dL)**	**Admission SBP (mmHg)**	**Time to Erythropiet in treatment**	**New infarction on follow up CT**	**CT- vasospasm related ischemia territory**	**mRS at ICU discharge**
	R MCA	R frontal	2	154	230	8	1	R MCA,R ACA	5
	R MCA	R frontal	2	217	101	2	1	R MCA	5
	R MCA	R frontal	2	217	101	11	0	0	5
	PICA r	R frontal	1	299	120	16	0	0	4
	R PCoA	R frontal	1	154	144	12	0	0	3
	ACoA	R frontal	1	96	144	9	0	0	2
	R MCA	R frontal	2	213	115	9	0	0	3
	ACoA	R frontal	1	184	120	6	1	L ACA, R MCA	5

R = right; L = left; H&H = Hunt-Hess Grade; SAH sum score grades the amount of blood in 10 basal cisterns and fissures (0 = no SAH, 1 = small SAH, 2 = moderate SAH, 3 = completely filled with SAH) by adding each of the 10 individual cistern scores (range 0–30); IVH sum score grades the amount of blood in the right and left lateral, third and fourth ventricle (0 = no blood, 1 = sedimentation, 2 = partly filled, 3 = completely filled) by adding each of the 4 individual ventricle scores (range 0–12); ACoA = anterior communicating artery; PCoA = posterior communicating artery; MCA = middle cerebral artery; SBP = systolic blood pressure; DCI = delayed cerebral ischemia; CT = computed tomography; SAH = subarachnoidal hemorrhage; ICH = intracerebral hemorrhage; IVH = intraventricular hemorrhage; mRS = modified Rankin Scale.

### **Effect of erythropoietin on physiologic variables**

**Figure 1:**
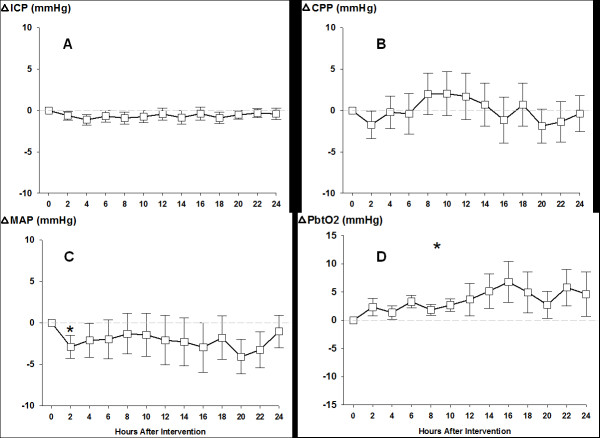
**Two-hourly averaged changes in cerebral perfusion pressure (CPP), mean arterial pressure (MAP), intracranial pressure (ICP) and brain tissue oxygen tension (PbtO2) following EPO (30.000 IU over 30 min) therapy, (mean ± SEM).** * P < 0.05.

Baseline CPP and MAP were 86 mmHg (IQR:77–95) and 99 mmHg (IQR:85–105), respectively, and ICP was within normal limits (7 mmHg, IQR:4–11). MAP decreased to 92 mmHg (IQR:82–101) 2 hours after intervention (P < 0.04) returning to baseline for the remaining 24 hours without significantly affecting CPP and ICP (Figure 1, Panel A-C).

### **Effect of erythropoietin on brain tissue oxygen tension and brain metabolism**

P_b_tO_2_ (baseline 29 mmHg, IQR:22–32) significantly increased over time by 4 ± 3 mmHg (mean increase compared to baseline) to a maximum of 7 ± 4 mmHg increase 16 hours after start of intervention (P < 0.05) (Figure 1, Panel D). All patients had an increase in P_b_tO_2_ at a certain point during the follow up time of 24 hours. Accounting for the EPO dose given (1^st^, 2^nd^, 3^rd^) per patient did not affect the observed result. Hemoglobin (baseline 9.5 mg/dL, IQR:9–11.5), body temperature (36.8° Celsius, IQR: 36,6-37.1) and SpO2 (96 %, IQR:95–97) remained stable and respirator settings were not changed during the 24 h-observation periods.

**Figure 2:**
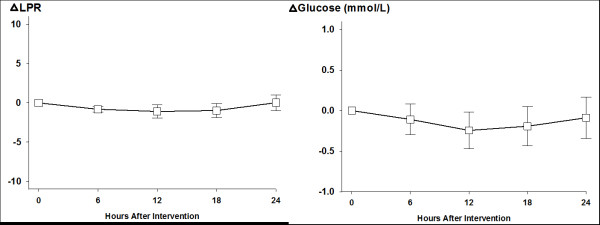
Six-hourly averaged change in lactat-pyruvate ratio (LPR) and brain tissue glucose concentration following EPO (30.000 IU over 30 min) therapy, (mean ± SEM).

Baseline brain metabolic data showed an increased median LPR (32, IQR:29–38) and slightly decreased brain glucose (1.1 mmol/L, IQR:0.8-2.8). Median brain lactate and pyruvate was 4.6 mmol/L (IQR:2.4-6.1) and 131 μmol/L (IQR:88–158), respectively. There was no significant change in brain lactate, pyruvate and brain glucose during the observation periods. (Figure 2, Panel A and B).

## Discussion

Our findings suggest that high-dose systemic EPO treatment increases brain tissue oxygen tension in patients with severe vasospasm after SAH. The present data are of potential importance because they support the hypothesis of a beneficial effect of high-dose systemic EPO beyond erythropoeisis on human brain tissue.

Vasospasm occurs in up to 70 % of aSAH patients, leads to decreased oxygen delivery with tissue hypoxemia, eventually resulting in brain tissue ischemia [1]. In in-vivo models of cerebral ischemia EPO and its receptor are highly expressed [11-13]. Experimental and clinical studies suggest a neuroprotective effect of EPO by decreasing inflammation, enhancing neurogenesis, inhibiting apoptosis and limiting SAH-induced oxidative damage especially in vulnerated brain tissue [9,10,14,15]. The effect of EPO on brain tissue oxygen tension may be multifactorial as P_b_tO_2_ is considered as marker of oxygen delivery, diffusion and consumption.

In an experimental model of ischemic tissue damage pretreatment with EPO improved microcirculation through increased arteriolar and venular blood flow with consecutive enhanced brain tissue oxygenation [16]. In this model, the beneficial effect of EPO was antagonized by blockade of NO synthase (NOS) activity, suggesting a link between EPO and NO-dependent vasodilatory effects [16], which has also been postulated in a rabbit SAH model of cerebral vasospasm [17,18]. After acute brain injury in humans, a strong correlation of P_b_tO_2_ and cerebral blood flow may exist in certain circumstances [19]. This may explain our findings of P_b_tO_2_-increase after EPO treatment, however, we did not measure cerebral blood flow in our patients to elaborate further on this finding. EPO may also enhance angiogenesis and thereby increase the amount of oxygen delivered to hypoxic tissue [20]. Proposed mechanisms include direct activation of endothelial cells by binding on the EPO-receptor or via activation of the vascular endothelial growth factor (VEGF)/VEGF receptor system [21]. In our patients, this hypothesis is unlikely as an increase in P_b_tO_2_ was observed within hours.

Another potential mechanism how EPO may increase PbtO2 is through its anti-inflammatory potency [22]. In vivo attenuation of the inflammatory response was achieved by EPO pretreatment in critically ischemic tissue with a significant effect after 5 hours [16]. Reduced metabolic demand and therefore oxygen consumption may eventually result in increased brain tissue oxygen tension with a latency of a few hours. Although we have not analyzed brain extracellular parameters of inflammation in our patients, the observed P_b_tO_2_ increase hours after EPO administration supports this hypothesis.

EPO did not improve brain metabolism, which replicates a previous study on 73 aSAH patients receiving 3 doses of EPO-alpha 500 IU/kg/day early after the initial bleed [8]. The moderate derangement in brain metabolism at baseline in the absence of brain tissue hypoxia in our patients suggests that an increase in PbtO2 may not necessarily translate into improved brain metabolism. The effect of EPO on brain homeostasis in the setting of aSAH patients with vasospasm-related ischemic pattern of brain metabolic crisis (LPR > 40 and Glucose < 0.7 mmol/L associated with P_b_tO_2_ <20 mmHg) would be of upmost interest. However, it seems important that EPO treatment has to be initiated in a timely fashion before ischemic damage occurs [11]. Elevation of cerebral LPR may also be explained by brain mitochondrial dysfunction [23], and EPO restored brain mitochondrial function in an experimental TBI model and may enhance cellular energy generation [10].

We used EPO as compassionate treatment in patients with severe cerebral vasospasm after aSAH following an institutional protocol. We carefully explore patients on underlying malignant diseases or risk of thromboembolism to limit this potential side effect of EPO [24]. Like in a previous trial including 80 aSAH patients, we used a high dose EPO regimen (total average dose of 90.000 IU) and did not observe any thromboembolic events [7].

Several limitations of the present study include a small sample size, a retrospective observational study design, a single institutional observation and lack of timed cranial CT scans. In addition, we did not routinely perform cerebral angiograms and the diagnosis of vasospasm was based on TCD studies. Therefore we want to emphasize, that in this pilot study we investigated the immediate physiologic response of high dose EPO on brain homeostasis in the setting of severe vasospasm. Moreover, we did not measure reticulocyte count in our patients, however hemoglobin and MAP remained stable after EPO treatment. Due to the small samples size a proper multivariate model could not be applied, however we did not observe changes in confounding factors of P_b_tO_2_, e.g. respirator settings. The ruptured aneurysm was located in the posterior circulation in 2 patients and the monitoring probes were placed in the ispsilateral right frontal white matter. We rarely consider invasive monitoring of regions in the occipital lobe or posterior fossa. Because both patients had a high SAH sum score, admission global cerebral edema and developed generalized vasospasm, the apparently remote distance between the vascular territory of the aneurysm bearing vessel and monitoring probes is of minor significance.

In this pilot study we did not gather information on cerebrovascular autoregulation and tissue enzyme activation which might also play a role in the EPO-related increases in P_b_tO_2_. Noteworthy, it has been shown that EPO can restore cerebral autoregulation after SAH [7,25] leading to enhanced cerebral blood flow and oxygen delivery to the brain. Also, EPO has been demonstrated to attenuate ischemia induced apoptotic cell death by modulation of the caspase cascade and may therefore influence P_b_tO_2_[16].

## Conclusions

These preliminary data suggest a beneficial effect of EPO in poor grade SAH patients with severe cerebral vasospasm. Further studies in larger cohorts are needed to evaluate the EPO effect on the long-term functional outcome of SAH patients.

## Competing interests

The authors declare that they have no competing interests.

## Authors` contributions

RH: concept, idea, analysis, writing, drafting, ES: writing, data collection. RB: idea, drafting, revising the manuscript. AC: angiogram, idea, drafting. MS: transcranial Doppler measurements, drafting. FS: insertion of multimodal neuromonitoring devices, drafting. GB: drafting. PL: drafting. MB: data collection. AZ: transcranial Doppler measurements, drafting. BP: idea, drafting. CT: drafting, editing, revising the manuscript. ES: idea, drafting, revising the manuscript. All authors have given final approval of the current version of this manuscript.

## Pre-publication history

The pre-publication history for this paper can be accessed here:

http://www.biomedcentral.com/1471-2377/12/32/prepub
